# Increased ectodomain shedding of cell adhesion molecule 1 as a cause of type II alveolar epithelial cell apoptosis in patients with idiopathic interstitial pneumonia

**DOI:** 10.1186/s12931-015-0255-x

**Published:** 2015-08-01

**Authors:** Azusa Yoneshige, Man Hagiyama, Takao Inoue, Takahiro Mimae, Takashi Kato, Morihito Okada, Eisuke Enoki, Akihiko Ito

**Affiliations:** Department of Pathology, Faculty of Medicine, Kinki University, Osaka, 589-8511 Japan; Department of Surgical Oncology, Research Institute for Radiation Biology and Medicine, Hiroshima University, Hiroshima, Japan

**Keywords:** α-shedding, Apoptosis, Emphysema, Nonspecific interstitial pneumonia, Paraffin section

## Abstract

**Background:**

Lung alveolar epithelial cell (AEC) apoptosis has attracted attention as an early pathogenic event in the development of idiopathic interstitial pneumonia (IIP); however, the causative mechanism remains unclear. Cell adhesion molecule 1 (CADM1) is an AEC adhesion molecule in the immunoglobulin superfamily. It generates a membrane-associated C-terminal fragment, αCTF, through protease-mediated ectodomain shedding, termed α-shedding. Increased CADM1 α-shedding contributes to AEC apoptosis in emphysematous lungs.

**Methods:**

Formalin-fixed, paraffin-embedded lung lobes (*n* = 39) from 36 autopsied patients with IIP were classified as acute IIP (*n* = 10), fibrosing-type nonspecific IIP (f-NSIP, *n* = 10), cryptogenic organizing IIP (*n* = 9), and usual IIP (*n* = 10). CADM1 expression in the lung sections was examined by western blotting and compared with control lungs (*n* = 10). The rate of CADM1 α-shedding was calculated as the relative amount of αCTF to full-length CADM1, and the full-length CADM1 level was estimated per epithelial cell by normalization to cytokeratin 7, a lung epithelial marker. Apoptotic AECs were detected by immunohistochemistry for single-stranded DNA (ssDNA). NCI-H441 and A549 human lung epithelial cells were transfected with small interfering RNA (siRNA) to silence CADM1 expression and analyzed by terminal nucleotide nick end labeling assays.

**Results:**

The rate of CADM1 α-shedding was higher in all IIP subtypes than in the control (*P* ≤ 0.019), and the full-length CADM1 level was lower in f-NSIP (*P* = 0.007). The α-shedding rate and full-length CADM1 level were correlated with each other (*P* = 0.015) and with the proportion of ssDNA-positive AECs (*P* ≤ 0.024). NCI-H441 cells transfected with siRNA exhibited a 61 % lower rate of expression of full-length CADM1 and a 17-fold increased proportion of apoptotic cells. Similar results were obtained with A549 cells.

**Conclusions:**

CADM1 α-shedding appeared to be increased in all four IIP subtypes and consequently contributed to AEC apoptosis by decreasing the full-length CADM1 level. This mechanism particularly impacted f-NSIP. The molecular mechanism causing AEC apoptosis may be similar between IIP and emphysema.

**Electronic supplementary material:**

The online version of this article (doi:10.1186/s12931-015-0255-x) contains supplementary material, which is available to authorized users.

## Introduction

Idiopathic interstitial pneumonias (IIPs) are a diverse group of diffuse parenchymal lung diseases of unknown etiology characterized by various degrees of acute or chronic inflammation and progressive fibrosis of the lung parenchyma. IIPs comprise several entities, including acute interstitial pneumonia (AIP), nonspecific interstitial pneumonia (NSIP), cryptogenic organizing pneumonia (COP), and usual interstitial pneumonia (UIP) or idiopathic pulmonary fibrosis (IPF) [1]. These entities share many features but are sufficiently different from one another in terms of their typical histological patterns. Accordingly, NSIP is further subclassified into cellular (c-NSIP) and fibrosing (f-NSIP) types [2]. This subtyping is of clinical importance, because the 5-year survival rate of c-NSIP is nearly 100 %, while that of f-NSIP is intermediate between those of c-NSIP and UIP, showing the worst rate among all of the entities [2]. Although the etiology of IIP remains enigmatic, there is a growing body of evidence supporting the theory that alveolar epithelial cell (AEC) injury and apoptosis contribute to the development and progression of IIP [3, 4]. In UIP, apoptotic AECs are found in areas of morphologically unaffected alveoli, suggesting that AEC apoptosis is a primary event prior to the onset of fibrosis and subsequently disrupts the basement membrane integrity, leading to various degrees of interstitial responses characterized by inflammatory cell infiltration and fibroblast recruitment [5]. Additionally, apoptosis in AIP is reportedly responsible for the resolution of type II AECs (AECIIs) during the early phase [6]. Molecular pathways that induce AEC apoptosis have been analyzed mainly in the setting of UIP or IPF. Intrinsic apoptotic markers (p53, p21, Bax, and caspase-3) and antiapoptotic markers (bcl-2) are increased and decreased, respectively, within AECs [7]. The extrinsic apoptotic factors Fas and Fas ligand are upregulated in AECs and infiltrating leukocytes, respectively [8]. Other mechanisms include increased oxidative stress products (hydrogen peroxide, myeloperoxidase, and nitric oxide) [9, 10], increased endoplasmic reticulum stress [11], and activation of hypoxia-inducible factor-1α [12]. Although AEC apoptosis is assumed to be a key event regardless of the IIP subtype, it remains unclear whether there is one AEC apoptosis mechanism common to all entities versus several mechanisms specific to each entity.

Cell adhesion molecule 1 (CADM1), also known as tumor suppressor in lung cancer 1 (TSLC1), is an intercellular adhesion molecule that belongs to the immunoglobulin (Ig) superfamily [13]. This membrane-spanning glycoprotein comprises three extracellular Ig-like domains, a single transmembrane region, and a short carboxy-terminal intracytoplasmic tail with a protein 4.1 interaction sequence and a PDZ type II domain-binding motif [13]. Various types of cells express CADM1, including pulmonary cells, biliary cells, renal tubular epithelial cells, neurons, mast cells, and pancreatic endocrine cells [14–17]. In epithelia, CADM1 is located on the lateral cell membrane and mediates neighboring cell–cell adhesion via *trans*-homophilic binding [18, 19]. Recent studies have shown that CADM1 expression is regulated by post-transcriptional mechanisms, including glycosylation and proteolytic cleavage, referred to as shedding [20, 21]. The ectodomain of CADM1 is cleaved at one of two sites, yielding two membrane-associated C-terminal fragments, αCTF and βCTF [21]. We recently identified CADM1 shedding as a key event in the development of pulmonary emphysema, a representative chronic obstructive pulmonary disease characterized by alveolar wall destruction and enlarged air spaces [22]. CADM1 ectodomain shedding rates increase in emphysematous lungs, and the mitochondrial localization of αCTF contributes to lung epithelial cell apoptosis [22].

In the present study, we evaluated the data of 39 patients diagnosed with IIP from the autopsy record archive of Kinki University Hospital and histologically classified them into four subtypes: AIP, f-NSIP, COP, and UIP. According to a previously described method [23], protein extracts were prepared from the autopsied lung paraffin sections and examined for CADM1 expression by Western blotting. We found that CADM1 ectodomain shedding increased and the full-length CADM1 level decreased in IIP lungs, with statistical significance being different among the subtypes. We examined whether these alterations in expression might be involved in AEC apoptosis in IIP, both *in vivo* and *in vitro*. This study identifies CADM1 shedding as a possible pathogenic event common to the four subtypes of IIP.

## Methods

### Antibodies

A rabbit anti-CADM1 polyclonal antibody directed against the C-terminal 15-amino acid peptide was generated in our laboratory as described previously [24]. Other primary antibodies used targeted cytokeratin 7 (CK7) (mouse monoclonal OV-TL 12/30; Dako, Glostrup, Denmark), single-stranded DNA (ssDNA) (rabbit IgG; Immuno-Biological Laboratories Co., Ltd., Gunma, Japan), thyroid transcription factor one (TTF1) (mouse monoclonal; Leica Biosystems, Nussloch, Germany), surfactant protein A (SP-A) (mouse monoclonal; Leica Biosystems), a cleaved form of caspase-3 (rabbit polyclonal; Cell Signaling Technology, Danvers, MA, USA), and glyceraldehyde 3-phosphate dehydrogenase (G3PDH) (Merck Millipore, Billerica, MA, USA). Peroxidase- and fluorophore-conjugated secondary antibodies were obtained from Amersham (Buckinghamshire, England) and Jackson ImmunoResearch (West Grove, PA, USA), respectively.

### Human samples

We reviewed the clinical records, autopsy records, and pathological specimens archived in Kinki University Hospital (Osaka, Japan) to identify autopsied patients who had been diagnosed with interstitial pneumonia and who had not received radiation therapy to the lung and had no clinical or histological indications of any diseases causing secondary interstitial pneumonia, such as collagen and infectious diseases. Autopsied patients who met these criteria were considered to have had IIP (*n* = 36 patients in the last 10 years, approximately six patients per year). The control group comprised 10 patients who had died of diseases without clinical or histological evidence of lung involvement in the last 10 years (approximately one patient per year). All patients were autopsied within a couple of hours after death. Both lungs were removed at autopsy and fixed with 10 % buffered formalin for several days. A subpleural segment of the middle portion of each lobe was then excised, embedded in paraffin, and cut serially into 20- and 3-μm-thick sections for protein extraction and histological examination, respectively. Two pathologists (E.E. and A.I.) examined the histologic sections and classified the IIP-affected lung lobes into AIP, f-NSIP, COP, and UIP groups according to the updated international multidisciplinary classification criteria. Three patients were given different diagnoses in the left and right lungs (see Additional file 1: Table S1). The Ethics Committee of Kinki University approved the experimental protocol (25-088).

### Protein extraction and western blot analysis

Protein was extracted from 20-μm-thick sections of paraffin-embedded lungs according to the method described by Rodríguez-Rigueiro et al. [25] with minor modifications [23]. Briefly, each section was deparaffinized by incubation in mineral oil at 95 °C for 2 mins, and the supernatant was removed by centrifugation at 11320 × *g*. The pellet was washed serially with phosphate-buffered saline and citrate-SDS buffer containing 200 mM Tris–HCl (pH 7.5), 200 mM NaCl, 5 % SDS, and 100 mM sodium citrate. Protein was extracted from the pellet by incubation at 100 °C for 20 mins and at 80 °C for 2 h in citrate-SDS buffer with occasional vortexing, then collected in the supernatant after centrifugation at 11320 × *g* for 15 mins at room temperature. The procedures of protein extraction from cultured cells and Western blot analyses were performed as described previously [18]. After Western blot transfer, the gels were stained with Coomassie Brilliant Blue (CBB) to indicate the amount of protein loading per lane. Immunoreactive band intensities were quantified using ImageJ software (National Institutes of Health, Bethesda, MD, USA), as described previously [26].

### Histological examination

Lung sections (3 μm thick) adjacent to those used for protein extraction were immunostained as described previously [18] using primary antibodies against CADM1 and ssDNA. Color reaction and counterstaining were performed using the peroxidase substrate 3-amino-9-ethylcarbazole (Vector Labs, Burlingame, CA, USA) and hematoxylin, respectively. AECIIs were morphologically identifiable as cuboidal cells >12 μm on a side lining the lumen of alveoli. AECIIs were often present in the alveolar space, in which case they were rounder in shape, similar to macrophages. When epithelial-like cell–cell contact was recognizable between two nonpigmented cells with a diameter >12 μm, the cells were regarded as AECIIs that had been detached from the alveolar lumen. Staining for ssDNA was found to be restricted to the nucleus, and the positive cells were AECIIs mainly and a few inflammatory blood cells. For each immunostained section, > 500 AECIIs were randomly selected and individually examined to determine whether they were positive for ssDNA. The proportion was calculated by dividing the number of ssDNA-positive AECIIs by the total cell number. The means and standard errors (SE) of the proportions were calculated for each group.

### Cell culture and transfection

NCI-H441 cells, a human lung epithelial cell line with characteristics of Clara cells, were purchased from the American Type Culture Collection (Rockville, MD, USA) in 2010 (Lot No. 58294188), and all experiments using this cell line were performed within 6 months after resuscitation. NCI-H441 cells were grown as described in our previous report [22]. A549 cells, a human lung epithelial carcinoma cell line, were described previously [22]. To silence the expression of CADM1, we constructed two p*Silencer*™ 4.1-CMV neo vectors (Applied Biosystems, Foster City, CA, USA), according to the manufacturer’s instructions using either of two small interfering RNAs (siRNAs) specifically targeting human CADM1, *i.e.*, siRNA-1, 5′-cgaaagacgtgacagtgat-3′(nt 275–293, numbered according to NM_001301043); and siRNA-2, 5′-ggccctatttagatgataa-3′ (nt 1579–1597), with reference to our previous report [27]. The negative control p*Silencer*™ 4.1-CMV neo vector (Applied Biosystems) contains a 55-base pair siRNA template with no significant homology to any mammalian gene. NCI-H441 and A549 cells were grown to 60–70 % confluence and transfected with either siRNA-encoding or control plasmid vector using Lipofectamine LTX and Plus reagents (Invitrogen, Carlsbad, CA, USA) according to the manufacturer’s instructions. After 48 h, the transfectants were subjected to Western blot analyses or assays for apoptosis detection.

### Detection of *in vitro* apoptosis

Terminal deoxynucleotidyl transferase-mediated dUTP nick end labeling (TUNEL) assays were conducted in NCI-H441 and A549 cells using the In Situ Cell Death Detection Kit (Roche Applied Science, Upper Bavarie, Germany) according to the manufacturer’s instructions, as described in our previous report [22]. Briefly, the cells were grown to 60–70 % confluence in μ-Dishes (ibidi, Verona, WI, USA) and transfected with the indicated vectors or left untransfected. After 48 h, the cells were fixed with 4 % paraformaldehyde, permeabilized with 0.1 % Triton X-100 in 0.1 % sodium citrate (pH 7.4), and incubated with the TUNEL reaction mixture containing terminal deoxynucleotidyl transferase and FITC-labeled dUTP for 1 h at 37 °C, followed by nuclear counterstaining with DAPI. Double-stained cultured cells were observed through a fluorescence microscope (Axio Observer D1; Carl Zeiss). When a cell exhibited TUNEL signals within the DAPI nuclear stain, the cell was deemed TUNEL-positive. The number of TUNEL-positive cells was counted among 500 NCI-H441or A549 cells. All measurements were performed in triplicate, and the mean and SE of the proportion of TUNEL-positive cells were calculated for each experimental group. The TUNEL assays were repeated three times with essentially similar results. Another set of H441 cells was fixed with 4 % paraformaldehyde, treated with 1.0 % bovine serum albumin and 0.3 % Triton X-100 in phosphate-buffered saline, and immunostained with an antibody against a cleaved form of caspase-3 and a Cy3-conjugated secondary antibody. The proportion of cleaved caspase-3-positive cells was calculated as described above for TUNEL assays.

### Double staining

Double immunofluorescence of lung sections was performed as described previously [22]. Sections were incubated with a mixture of antibodies against ssDNA and SP-A, and then reacted with Alexa Flour 488- and 594-conjugated secondary antibodies. Double staining of lung sections for two apoptotic markers ssDNA and TUNEL was performed as described previously [22]. Sections were first stained with a Click-iT Plus TUNEL Assay Kit containing an Alexa Flour 488-conjugated secondary antibody (Molecular Probes, Eugene, OR, USA) according to the manufacturer’s instructions, and were next immunostained with the anti-ssDNA antibody, followed by reaction with an Alexa Flour 594-conjugated secondary antibody. The percentage of double-positive cells among 200 ssDNA-positive AECIIs was calculated for each case.

### Statistical analysis

Clinical variables of autopsied patients were assessed between any two groups using the Steel–Dwass test for age and the *χ*^2^ test for sex. Statistical differences between any two groups were analyzed using the Steel–Dwass test to assess the quantified Western blot data and the proportions of AECIIs. Correlations were analyzed using Spearman’s rank test. For in vitro studies, the quantified Western blot data and TUNEL-positive cell proportions were compared using Student’s *t*-test. A *P*-value ≤ 0.05 was considered statistically significant.

## Results

### Increased ectodomain shedding of CADM1 in IIP lungs

**Fig. 1:**
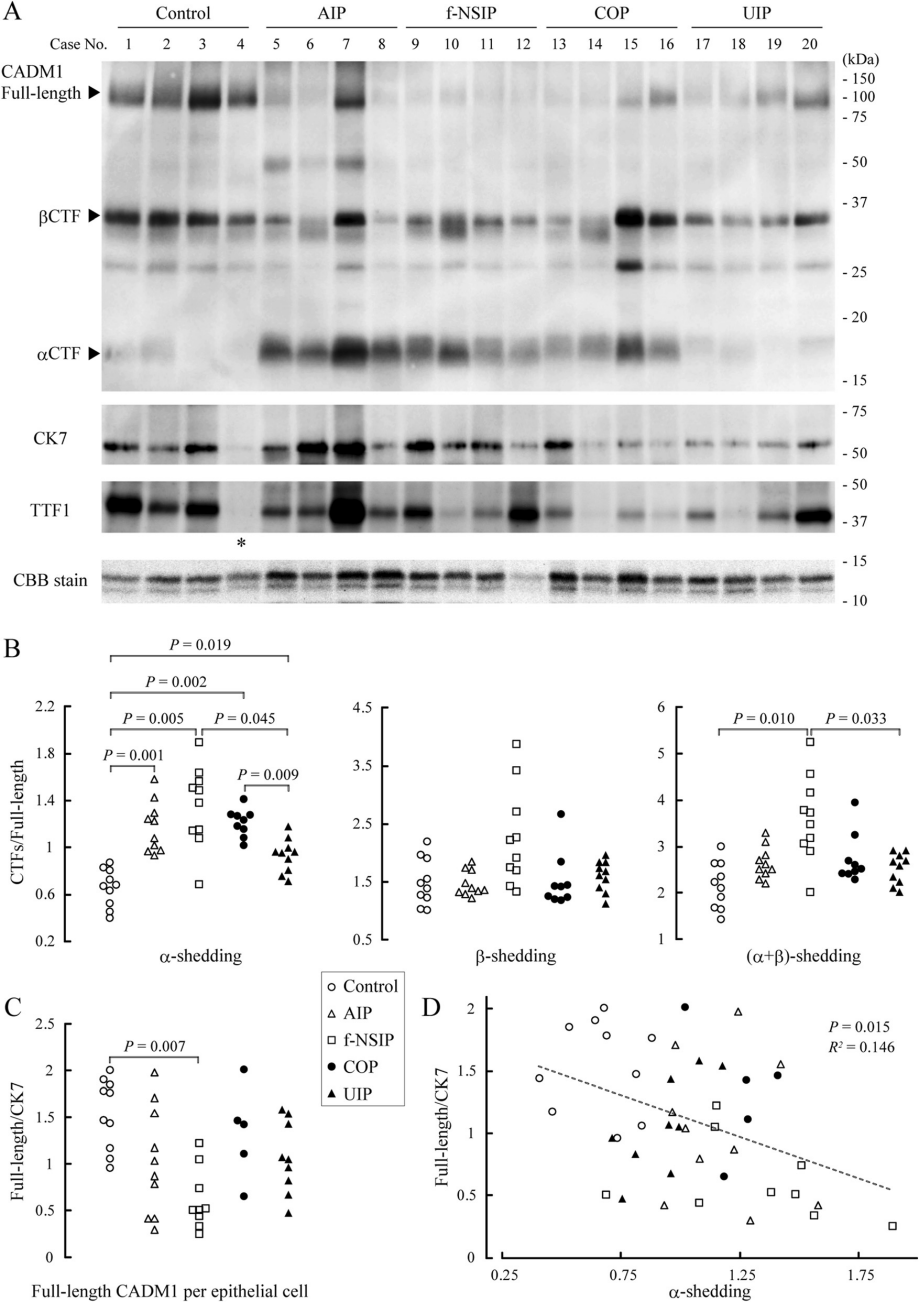
Western blot analysis of CADM1 expression in IIP lungs. **a** Protein was extracted from control and IIP lung sections and subjected to Western blot analyses using an anti-CADM1 antibody. Cases are numbered as in Additional file 1: Table S1. Results of the remaining cases are shown in Additional file 2: Figure S1. Arrowheads indicate bands corresponding to the full-length, αCTF, and βCTF forms of CADM1. The blot was reprobed with an anti-CK7 and anti-TTF1 antibody to estimate the number of epithelial cells and AECIIs, respectively. An asterisk indicates the sample that did not gave a clear immunoreactive band for TTF1. After Western blot transfer, the gels were stained with CBB to indicate the amount of protein loading per lane. **b** CADM1 ectodomain shedding rates (amount of CTFs relative to full-length CADM1) and **c** full-length CADM1 level per lung epithelial cell (relative to CK7) in each patient sample are plotted as dots. Statistical significance between two groups was analyzed using the Steel–Dwass test. *P*-values ≤ 0.05 are shown. **d** The full-length CADM1 level per epithelial cell with the CADM1α-shedding rate is shown in a scatter plot, and the dot distribution approximates a linear function (dotted line). Correlations and statistical significance were analyzed using Spearman’s rank test. *R* ^*2*^ and *P*-values are shown

Thirty-nine lung lobes from 36 autopsied patients with IIP were classified into four groups: AIP (*n* = 10), f-NSIP (*n* = 10), COP (*n* = 9), and UIP (*n* = 10). The patients’ characteristics are summarized in Additional file 1: Table S1. Including the control group, there was no difference in age or sex between any two groups. We extracted protein from peripheral lung parenchyma sections and analyzed the extracts by Western blotting using a CADM1 antibody (Fig. 1a and Additional file 2: Figure S1). Consistent with our previous report [22], the CADM1 antibody detected three forms of CADM1 approximately 100, 35, and 18 kDa in size, corresponding to full-length CADM1, βCTF, and αCTF, respectively (Fig. 1a and Additional file 2: Figure S1). The CADM1 shedding rates were calculated as the band intensities of αCTF, βCTF, and αCTF + βCTF relative to that of full-length CADM1 and designated the α-, β-, and (α + β)-shedding rates, respectively. All four IIP groups had higher α-shedding rates than those of the control group, while there was no significant difference in the β-shedding rate between any two groups (Fig. 1b). The (α + β)-shedding rate in f-NSIP lungs was the highest, with significant differences from both the control and UIP groups (Fig. 1b).

We previously reported that CADM1 distributed exclusively to bronchial and alveolar epithelial cells in the human peripheral lung parenchyma [14]. Thus, epithelial cells were considered to be the major cell source of CADM1. The Western blots were reprobed using an antibody to CK7 (a lung epithelial marker) [28], and full-length CADM1 expression per epithelial cell was estimated by normalizing the band intensity of CADM1 to CK7 (Fig. 1a and Additional file 2: Figure S1). Some samples did not give a clear single band for CK7 (Additional file 2: Figure S1) and were thus excluded from further analyses (control, *n* = 10; AIP, *n* = 10; f-NSIP, *n* = 9; COP, *n* = 5; and UIP, *n* = 9) (Additional file 1: Table S1). On average, the full-length CADM1 level per epithelial cell was lower in all four IIP groups than in the control group, but the decrease was statistically significant only in the f-NSIP group (Fig. 1c).

A scatter plot of all cases and Spearman correlation analyses revealed that the full-length CADM1 level per epithelial cell was inversely correlated with the α-shedding rate (Fig. 1d), suggesting that increased α-shedding contributed to the decreased levels of full-length CADM1 in lung epithelial cells of patients with IIP.

### Correlation between CADM1 shedding and AECII apoptosis in IIP

**Fig. 2:**
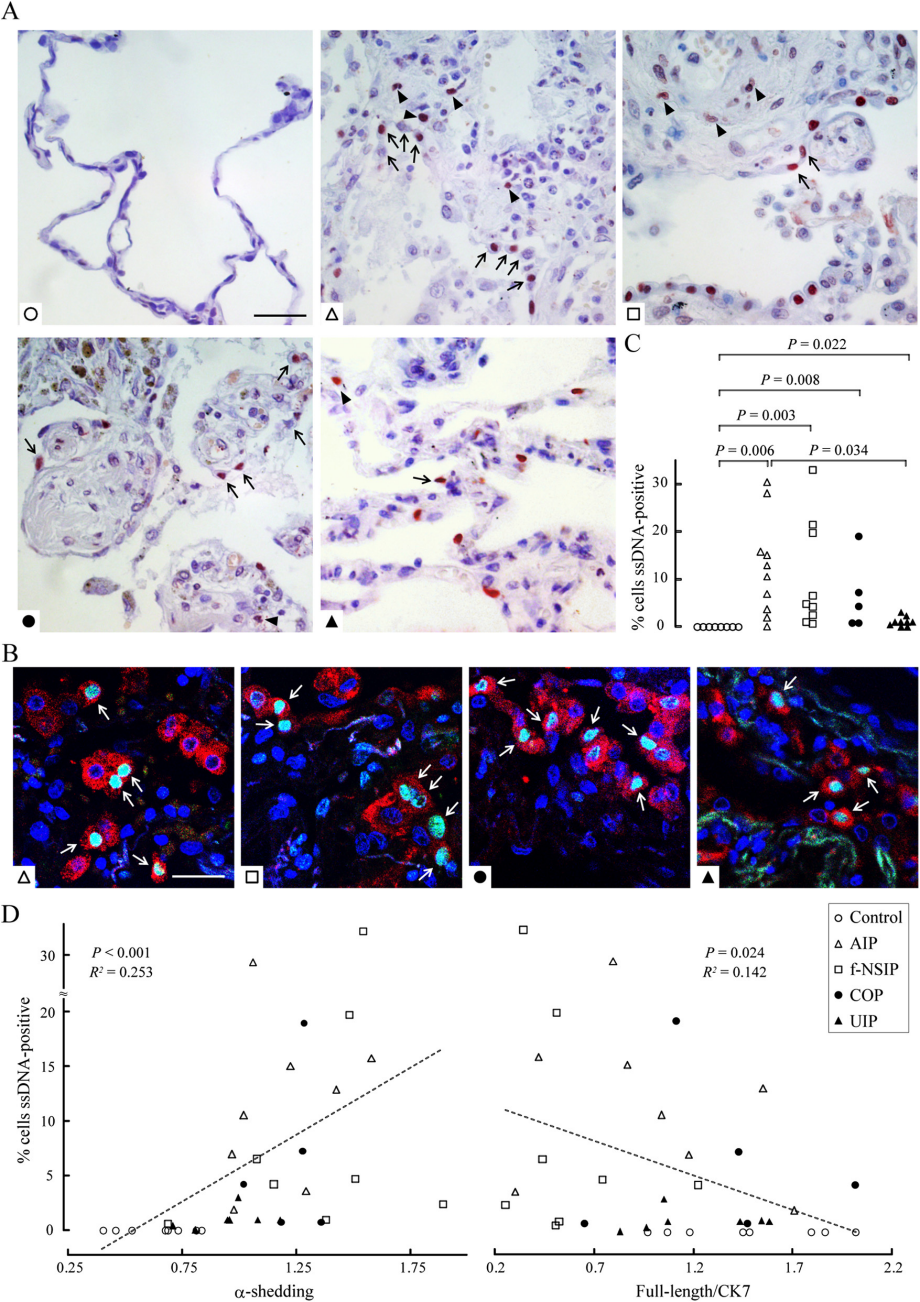
Correlation between AECII apoptosis and CADM1 ectodomain shedding in IIP. **a** Control and IIP lung sections were stained immunohistochemically with an anti-ssDNA antibody and counterstained with hematoxylin. A representative photomicrograph for each group is shown. Arrows indicate AECIIs detached from the alveolar wall. Arrowheads indicate ssDNA-positive inflammatory blood cells, as opposed to AECIIs. Bar = 50 μm. **b** IIP lung sections were double immunostained with antibodies against ssDNA (green) and SP-A (red), and counterstained with DAPI (blue). A representative result for each subtype is shown as a picture where the three fluorescence images are merged. Arrows indicate cells double positive for ssDNA and SP-A. Bar = 25 μm. **c** Proportions of ssDNA-positive AECIIs in each patient sample are plotted as dots, and statistical significance between two groups was analyzed using the Steel–Dwass test. *P*-values ≤ 0.05 are shown. **d** The proportion of ssDNA-positive AECIIs with the CADM1 α-shedding rate (*left*) and the full-length CADM1 level per epithelial cell (*right*) are shown in a scatter plot. In each graph, the dot distribution approximates a linear function (dotted lines). Correlations and statistical significance were analyzed using Spearman’s rank test. *R* ^*2*^ and *P*-values are shown

The lung sections were immunostained with an antibody targeting ssDNA to detect apoptotic cells [29] and counterstained with hematoxylin, which allowed morphological identification of AECIIs (Fig. 2a). Apoptotic AECIIs were clearly detected as cells double positive for ssDNA and SP-A, a AECII marker, by double immunofluorescence in all four IIP subtypes (Fig. 2b). The proportion of ssDNA-positive AECIIs was practically zero in the control lungs, whereas it was significantly increased in all four IIP groups (Fig. 2c). The scatter plot and Spearman correlation analyses revealed that this proportion was correlated positively with the α- and (α + β)-shedding rates and inversely with the full-length CADM1 level per epithelial cell (Fig. 2d, and Additional file 3: Figure S2, A and B). In IIP lungs, AECIIs were often detached from the alveolar wall (Fig. 2a). Because some were ssDNA-positive but others ssDNA-negative, these detached cells were considered to be dead due to apoptosis including anoikis or other mechanisms including necrosis [30]. Some AECIIs were both ssDNA-positive and detached.

**Fig. 3:**
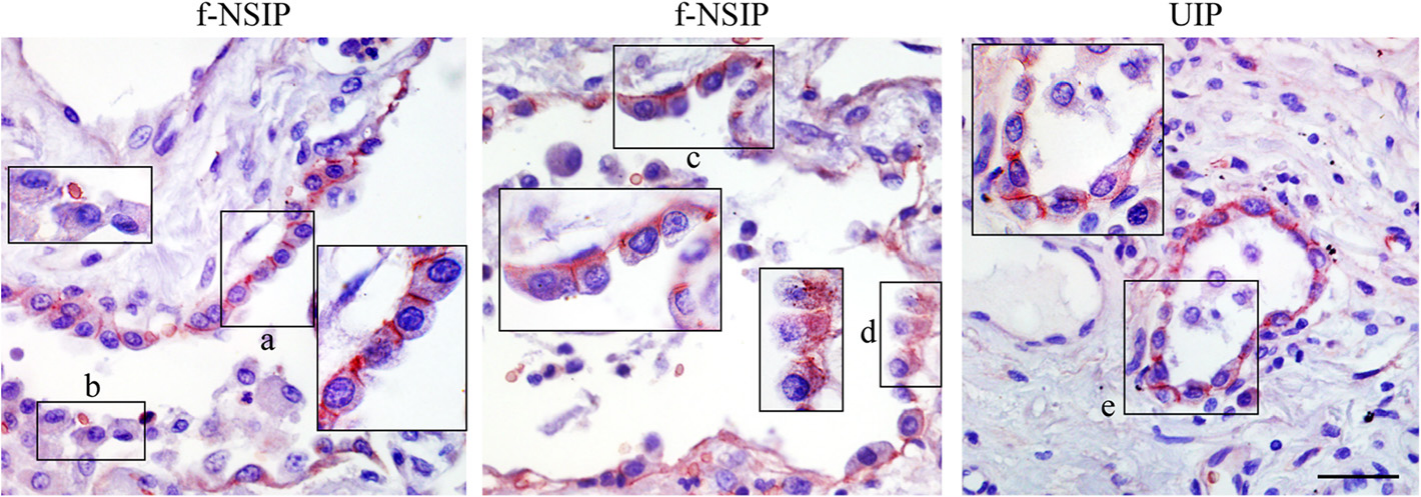
Immunohistochemical analysis of CADM1 in IIP lungs. IIP lung sections were stained immunohistochemically with an anti-CADM1 antibody and counterstained with hematoxylin. Representative results for f-NSIP and UIP are shown. Boxed areas are enlarged to depict different subcellular localizations of CADM1 in AECIIs that are lining the alveolar lumen (*a*, *c* and *e*), detaching (*d* and *e*), or detached (*b*) from the alveolar wall. Bar = 50 μm. Results of the other subtypes are shown in Additional file 4: Figure S3

CADM1 expression in IIP lungs was examined by immunohistochemistry. In AECIIs lining the alveolar lumen, CADM1 was detected primarily on the lateral membrane, which is the proper localization for full-length CADM1 [14], regardless of the IIP subtype (Fig. 3 and Additional file 4: Figure S3). In contrast, CADM1 membranous staining rarely occurred in AECIIs that were detached or detaching from the wall; instead, intracytoplasmic staining was often noticeable (Fig. 3). This probably reflects decreased full-length CADM1 levels and increased production of αCTF due to increased α-shedding, as shown in emphysematous lungs [22]. AECIIs with this staining pattern were predominantly observed in f-NSIP lungs. These results provide histologic evidence of a relationship of AECII apoptosis with increased α-shedding and decreased full-length CADM1 levels. To gain further evidence for this relationship, we reprobed the Western blot of lung tissues with an antibody against TTF1, a AECII marker (Fig. 1a and Additional file 2: Figure S1). When the full-length CADM1 level was normalized to TTF1, it had a propensity to inversely correlate with the percentage of ssDNA-positive AECIIs but did not reach statistical significance (Additional file 3: Figure S2C), probably due to varying proportions of AECIIs in individual lung sections. In addition, we assessed AECII apoptosis by double immunofluorescence using TUNEL as a second apoptosis marker in two or three cases for each IIP subtype (Additional file 5: Figure S4). More than 80 % of ssDNA-positive AECIIs were positive for TUNEL, confirming the validity of ssDNA immunohistochemistry.

### Increased apoptosis of lung epithelial cells by CADM1-targeting siRNA

**Fig. 4:**
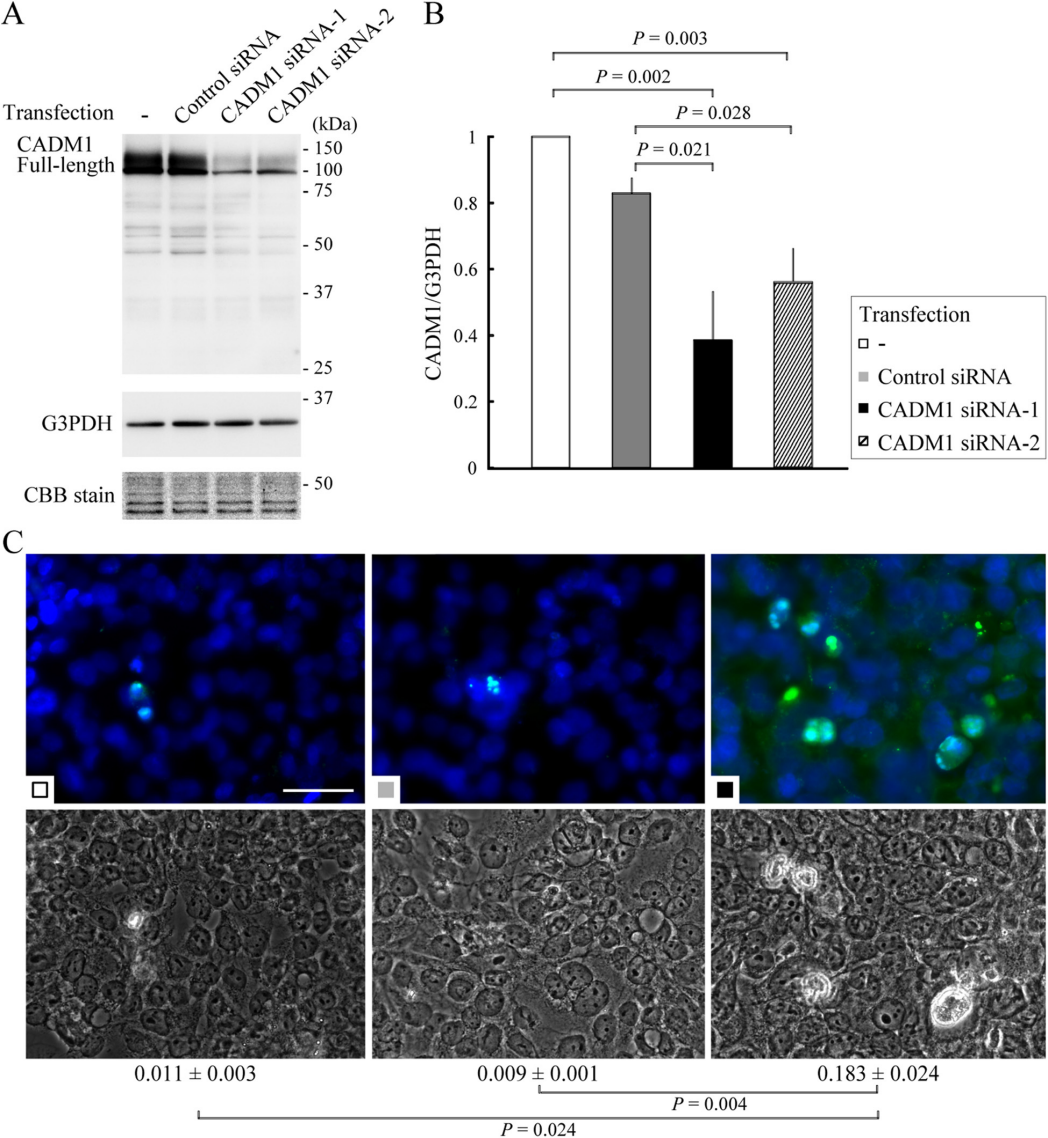
Increased apoptosis of NCI-H441 cells transfected with an siRNA targeting CADM1. **a** NCI-H441 cells were left untreated (–) or transfected with control or CADM1-targeting siRNA (siRNA-1 and -2). After 48 h, CADM1 expression was examined by Western blot analyses. The blot was reprobed using an anti-G3PDH antibody to normalize full-length CADM1 levels to G3PDH. After Western blot transfer, the gels were stained with CBB to indicate the amount of protein loading per lane. **b** The relative CADM1 levels were further normalized to a value of 1 in untreated cells, to which the CADM1 levels in transfectants were then normalized. The mean ± SE full-length CADM1 levels were calculated from triplicate experiments for each cell type, and statistical significance was analyzed using Student’s *t*-test. *P*-values ≤ 0.05 are shown. **c** Another set of NCI-H441 cells untreated and transfected with either control or siRNA-1 vector was analyzed by TUNEL assays. In the upper column, TUNEL (green) and DAPI (blue) fluorescent images were merged. The differential interference contrast images are shown on the lower. Bar = 50 μm. The mean proportions of TUNEL-positive cells and the SE were indicated under the panels. *P*-values ≤ 0.05 by Student’s *t*-test are shown. Results of siRNA-2 transfectants are shown in Additional file 6: Figure S5

NCI-H441 human lung epithelial cells were transfected with a plasmid vector encoding either specific siRNA targeting CADM1 (siRNA-1 and -2) or control siRNA. After 48 h, the cells were subjected to Western blot analyses using antibodies to CADM1 and G3PDH. Full-length CADM1 levels were normalized to G3PDH levels. CADM1-targeting siRNA-1 and -2 reduced the level of full-length CADM1 by approximately 61 and 43 %, respectively, while control siRNA had no effect on the level (Fig. 4a and b). Another set of transfectants was examined for frequency of apoptosis by TUNEL assays. The percentage of TUNEL-positive cells was around 1 % in untreated cells and control siRNA transfectants but approximately 18 and 5 % in CADM1-targeting siRNA-1 and -2 transfectants (*P* ≤ 0.024) (Fig. 4c and Additional file 6: Figure S5A). Consistent results were obtained when apoptosis was detected by immunohistochemistry using an antibody against cleaved caspase-3, a key apoptosis effector [31] (Additional file 6: Figure S5B). Experiments were repeated using A549 human lung epithelial cells, with results essentially similar to those of H441 cells (Additional file 7: Figure S6).

## Discussion

In the present study, we examined CADM1 expression in four histologic subtypes of IIP and found that CADM1 α-shedding was increased in all four subtypes and that the full-length CADM1 level was decreased in f-NSIP. We also found that the α- or (α + β)-shedding rate and the full-length CADM1 level were correlated with one another and with the proportion of ssDNA-positive AECIIs, suggesting a significant contribution of CADM1 shedding to AECII apoptosis in IIP lungs. Two mechanisms for these phenomena are proposed. One involves αCTF, an α-shedding product of CADM1. As we previously showed in emphysematous lungs, once αCTF is produced in AECIIs, it accumulates preferentially in the mitochondria and consequently depolarizes the mitochondrial membrane potential, resulting in activation of the mitochondrial apoptotic pathway in AECIIs [22]. As shown in Fig. 3, CADM1 was often detected in the cytoplasm, but not on the cell membrane, of detaching AECIIs, suggesting accumulation of αCTF in the mitochondria. The notion that the mitochondrial apoptotic pathway is activated in IIP lung epithelial cells was described as early as 2002 by Kuwano et al. [32]. The present study reinforces this notion and proposes αCTF as a key molecule in the activation of this pathway. Another mechanism involves the decrease in full-length CADM1 levels as a result of increased CADM1 shedding. As evidenced by the siRNA transfection experiment, decreased full-length CADM1 expression in lung epithelial cells appeared to be associated with increased apoptosis. We previously performed similar experiments and reported that downregulation of CADM1 by siRNA abrogates the formation of epithelial cell structure, suggesting an essential role for full-length CADM1 in the maintenance of epithelial cell polarity [33]. Because loss of cell polarity can trigger apoptosis to eliminate damaged epithelial cells [34, 35], full-length CADM1 downregulation supposedly causes AECII apoptosis through loss of epithelial polarity. Considering the present Western blot data, these two mechanisms may contribute differently to the pathogenesis of individual IIP subtypes; the αCTF mechanism profoundly impacts AIP, f-NSIP, and COP and relatively mildly impacts UIP, while the full-length CADM1 mechanism acts particularly on f-NSIP.

Among the four IIP subtypes examined, f-NSIP was notable, because it exhibited the highest shedding rates and expressed the lowest level of full-length CADM1 per epithelial cell, suggesting strong involvement of CADM1 shedding in the pathogenesis of f-NSIP. NSIP has some degree of overlap with a variety of other IIP subtypes [2]. Yang et al. [36] examined the gene expression profiles of NSIP and UIP and concluded that the two entities are similar transcriptionally. However, they identified eight transcripts that best differentiate the two, including the serine protease cathepsin G gene, which was upregulated in NSIP [36]. Another study by Takahashi et al. [37] reported that matrix metalloproteinase-2 (MMP-2) mRNA levels are higher in NSIP than in UIP. The fact that these two proteases are released from neutrophils and macrophages [38, 39] is consistent with our clinical experiences showing that NSIP behaves as a more inflammatory process, in distinct contrast to UIP [1, 2]. Both cathepsin G and MMP-2, once released, are executers of ectodomain shedding of Ig superfamily adhesion molecules, such as ICAM-1 or 5 and VCAM-1 expressed on bone marrow cells or neurons [40–42]. The particularly increased CADM1 shedding in f-NSIP may be attributable to the high expression levels of these proteases.

The f-NSIP group also differed substantially from the UIP group with respect to the incidence of AECII apoptosis. Some previous studies have demonstrated that in UIP, AECII apoptosis occurs actively in morphologically unaffected lung parenchyma around the remodeled fibrotic lesion, but only occasionally within the fibrotic lesion, where the percentage of apoptotic AECIIs is approximately 1–5 % (in contrast, it is practically 0 % in the normal lung) [5, 32, 43]. These values are consistent with our histological findings in the UIP fibrotic lesions and control lungs. Only a few referable studies on NSIP are available; these studies reported that 1-2 % of AECIIs were apoptotic in NSIP (whether the cellular or fibrosing subtype was not specified) [32, 43]. Compared with this estimation, our measured values (10.4 ± 3.8 %) are quite high. This may represent a limitation of the present study, as we analyzed only autopsied cases. This sampling method might have introduced significant bias in the patient pool compositions. In fact, six of ten patients in the f-NSIP group and three of nine patients in the COP group died of IIP itself, although these IIP entities reportedly have fairly good 5-year survival rates [2]. Our f-NSIP and COP groups may have comprised more patients with severe pathophysiology. Another interpretation of the present results is that active AECII apoptosis may contribute to treatment resistance and poor prognoses in patients with f-NSIP and COP.

Among the four subtypes, the incidence of AECII apoptosis was the highest in AIP, which is consistent with several past studies [6, 44]. Serial activation of macrophages and neutrophils has long been believed to be an important pathogenic process for AECII injury in AIP, but how these cells act on AECIIs is not entirely clear. When activated, these inflammatory cells potently release a variety of products, including proteases such as cathepsin G and MMP-2, as described above. Inflammatory cell infiltration may cause local increases in proteolytic activities and thereby promote AECII apoptosis by increasing CADM1 shedding in AIP lungs.

In conclusion, the present study demonstrated a close link between increased CADM1 ectodomain shedding and increased AECII apoptosis in IIP and suggests a pathophysiologic commonality between IIP and pulmonary emphysema at the molecular level. Following our previous characterization of CADM1 as a human pancreatic islet cell adhesion molecule involved in hormone secretion [18], we recently found that CADM1 shedding was increased in type two diabetes mellitus pancreata and involved in β cell apoptosis [23]. Taken together, our findings indicate that CADM1 shedding may trigger apoptosis in cells that require CADM1 to fulfill their proper function. Although the precise mechanisms of increased CADM1 shedding in individual diseases remain to be clarified, it is noteworthy that ectodomain shedding is a proteolytic process mediated by proteases. As shown in pulmonary emphysema [22], protease-over-antiprotease activity imbalance may be a precedent key event in lesions in which CADM1-expressing cell apoptosis occurs. Although this study identified the molecular distinctions among IIP subtypes, it also suggests that IIP, regardless of the subtype, may share a pathogenic molecular process with more common diseases, such as type two diabetes mellitus and pulmonary emphysema.

## Additional files

Additional file 1: Table S1. Characteristics of autopsied patients (DOCX 26 kb) Additional file 2: Figure S1. Western blot analysis of CADM1 expression in IIP lungs. Protein was extracted from control and IIP lung sections and subjected to Western blot analyses using an anti-CADM1 antibody. Cases are numbered as in Additional file 1: Table S1. Arrowheads indicate bands corresponding to the full-length, αCTF, and βCTF forms of CADM1. The blot was reprobed with an anti-CK7 and anti-TTI1 antibody to estimate the number of epithelial cells and AECIIs, respectively. Asterisks indicate the samples that did not gave a clear immunoreactive band for CK7 or TTF1. After Western blot transfer, the gels were stained with CBB to indicate the amount of protein loading per lane. (TIFF 1277 kb) Additional file 3: Figure S2. Correlations of the full-length CADM1 level per epithelial cell or AECII apoptosis with (α + β)-shedding rate in IIP. (A) The full-length CADM1 level per epithelial cell with the CADM1 (α + β)-shedding rate are shown in a scatter plot. (B and C) The proportion of ssDNA-positive AECIIs with the CADM1 (α + β)-shedding rate (B) and the full-length CADM1 level normalized to TTF1 (C) are shown in scatter plots. In each graph A, B and C, the dot distribution approximates a linear function (dotted lines). Correlations and statistical significance were analyzed using Spearman’s rank test. *R* ^*2*^ and *P*-values are shown. (TIFF 208 kb) Additional file 4: Figure S3. Immunohistochemical analysis of CADM1 in IIP lungs. IIP lung sections were stained immunohistochemically with an anti-CADM1 antibody and counterstained with hematoxylin. Representative results for control lungs, AIP and COP are shown. Boxed areas are enlarged to depict different subcellular localizations of CADM1 in epithelial cells that are lining the alveolar lumen (a, b and f), detaching (e and g), or detached (c and d) from the alveolar wall. Bar = 50 μm. (TIFF 2572 kb) Additional file 5: Figure S4. Double staining of IIP lungs for two apoptosis markers, ssDNA and TUNEL. IIP lung sections from the cases indicated were stained serially using the TUNEL methods (green) and ssDNA immunofluorescence (red), and then reacted with DAPI (blue) to stain the nucleus. Representative results from the cases indicated by asterisks are shown as pictures where the three fluorescence images are merged. White signals indicate the nuclei double positive for ssDNA and TUNEL. Some nuclei are single positive for either marker depicted by arrows. The proportions of double-positive cells among ssDNA-positive AECIIs (concordance rate) are shown in the rightmost column. Bar = 50 μm. (TIFF 1437 kb) Additional file 6: Figure S5. Increased apoptosis of NCI-H441 cells transfected with a CADM1 siRNA-2 vector. (A) NCI-H441 cells were untreated or transfected with either control or CADM1 siRNA-2 vector, as described in Fig. 4, and then were analyzed by TUNEL assays. In the upper column, TUNEL (green) and DAPI (blue) fluorescent images were merged. The differential interference contrast images are shown on the lower. (B) Another set of NCI-H441 cells untreated and transfected with either control or CADM1 siRNA-2 vector was stained by immunofluorescence for cleaved caspase-3. In the upper column, cleaved caspase-3 (red) and DAPI (blue) fluorescent images were merged. The differential interference contrast images are shown on the lower. The mean proportions of TUNEL-positive cells and the SE were indicated under the panels. *P*-values ≤ 0.05 by Student’s *t*-test are shown. Bar = 50 μm. (TIFF 3101 kb) Additional file 7: Figure S6. Increased apoptosis of A549 cells transfected with an siRNA targeting CADM1. (A) A549 cells were left untreated (–) or transfected with control or CADM1-targeting siRNA (siRNA-2). After 48 h, CADM1 expression was examined by Western blot analyses. The blot was reprobed using an anti-G3PDH antibody to normalize full-length CADM1 levels to G3PDH. After Western blot transfer, the gels were stained with CBB to indicate the amount of protein loading per lane. (B) The relative CADM1 levels were further normalized to a value of 1 in untreated cells, to which the CADM1 levels in transfectants were then normalized. The mean ± SE full-length CADM1 levels were calculated from triplicate experiments for each cell type, and statistical significance was analyzed using Student’s *t*-test. *P*-values ≤ 0.05 are shown. (C) Another set of A549 cells untreated and transfected with either control or siRNA-2 vector was analyzed by TUNEL assays. In the upper column, TUNEL (green) and DAPI (blue) fluorescent images were merged. The differential interference contrast images are shown on the lower. Bar = 50 μm. The mean proportions of TUNEL-positive cells and the SE were indicated under the panels. *P*-values ≤ 0.05 by Student’s *t*-test are shown. (TIFF 1716 kb)
