# Supplementary material for: Increased ectodomain shedding of cell adhesion molecule 1 as a cause of type II alveolar epithelial cell apoptosis in patients with idiopathic interstitial pneumonia
Source: Respir Res. 2015 Aug 1;16:90. doi: 10.1186/s12931-015-0255-x (PMC4531801; doi:10.1186/s12931-015-0255-x)
Supplement: Additional file 1: Table S1. — Characteristics of autopsied patients (DOCX 26 kb) [file 12931_2015_255_MOESM1_ESM.docx]

Supplementary Table S1 Characteristics of autopsied patients

| Group | | Case No. | | Age | | Sex | |  | | Primary cause of death | | Date of death (y/m/d) | | Lobe examined | | CK7  expression | | TTF1  expression | |
| --- | --- | --- | --- | --- | --- | --- | --- | --- | --- | --- | --- | --- | --- | --- | --- | --- | --- | --- | --- |
| Control | 1 | | 70 | | F | |  | | Dilated cardiomyopathy | | 06/6/14 | | - | | D | | D | |  |
|  | 2 | | 72 | | F | |  | | Hepatocellular carcinoma | | 07/4/26 | | - | | D | | D | |  |
|  | 3 | | 69 | | M | |  | | Esophageal carcinoma | | 12/6/22 | | - | | D | | D | |  |
|  | 4 | | 43 | | M | |  | | Infectious colitis | | 11/4/23 | | LU | | D | | N | |  |
|  | 21 | | 76 | | M | |  | | Hepatocellular carcinoma | | 09/1/4 | | - | | D | | D | |  |
|  | 22 | | 75 | | F | |  | | Aortic valve stenosis | | 11/11/11 | | RL | | D | | D | |  |
|  | 23 | | 64 | | F | |  | | Amyotrophic lateral Sclerosis | | 09/6/10 | | - | | D | | N | |  |
|  | 24 | | 61 | | F | |  | | T cell lymphoma | | 10/6/16 | | - | | D | | D | |  |
|  | 25 | | 73 | | M | |  | | Hepatocellular carcinoma | | 06/9/14 | | - | | D | | D | |  |
|  | 26 | | 70 | | M | |  | | Liver cirrhosis | | 07/3/4 | | RM | | D | | D | |  |
|  |  | |  | |  | |  | |  | |  | |  | |  | |  | |  |
| AIP | 5 | | 69 | | M | |  | | Gastric carcinoma | | 09/12/3 | | LU | | D | | D | |  |
|  | 6 | | 45 | | M | |  | | Liver cirrhosis | | 11/1/23 | | - | | D | | D | |  |
|  | 7 | | 57 | | M | |  | | Cholangiocarcinoma | | 11/5/14 | | RL | | D | | D | |  |
|  | 8 | | 85 | | F | |  | | IIP | | 07/4/17 | | - | | D | | D | |  |
|  | 27 | | 53 | | F | |  | | Acute myeloid leukemia | | 08/9/13 | | - | | D | | D | |  |
|  | 28^a^ | | 82 | | M | |  | | Congestive heart failure | | 10/2/3 | | LU | | D | | D | |  |
|  | 29 | | 73 | | M | |  | | Non-Hodgkin lymphoma | | 09/5/6 | | - | | D | | D | |  |
|  | 30 | | 61 | | M | |  | | IIP | | 08/8/18 | | LU | | D | | N | |  |
|  | 31 | | 73 | | M | |  | | Post-operative lung edema | | 09/9/19 | | RU | | D | | D | |  |
|  | 32^b^ | | 67 | | M | |  | | IIP | | 07/1/5 | | RL | | D | | D | |  |
|  |  | |  | |  | |  | |  | |  | |  | |  | |  | |  |
| f-NSIP | 9 | | 75 | | M | |  | | Rectal carcinoma | | 06/7/5 | | RU | | D | | D | |  |
|  | 10 | | 83 | | M | |  | | IIP, left lung carcinoma | | 07/3/8 | | RM | | D | | D | |  |
|  | 11 | | 76 | | M | |  | | Brain infarction | | 10/5/30 | | - | | D | | D | |  |
|  | 12^c^ | | 65 | | F | |  | | IIP | | 08/5/13 | | LU | | D | | D | |  |
|  | 33 | | 70 | | M | |  | | IIP | | 07/4/29 | | RM | | N | | N | |  |
|  | 34 | | 71 | | M | |  | | Esophageal carcinoma | | 07/1/12 | | RU | | D | | N | |  |
|  | 35 | | 58 | | F | |  | | IIP | | 11/2/10 | | - | | D | | D | |  |
|  | 36 | | 19 | | F | |  | | Dilated cardiomyopathy | | 08/6/20 | | RM | | D | | D | |  |
|  | 37 | | 79 | | M | |  | | IIP, Myelodysplastic syndrome | | 08/2/21 | | LL | | D | | D | |  |
|  | 38 | | 62 | | M | |  | | IIP | | 11/4/14 | | RM | | D | | D | |  |
|  |  | |  | |  | |  | |  | |  | |  | |  | |  | |  |
| COP | 13 | | 83 | | M | |  | | Acute pancreatitis | | 06/8/4 | | - | | D | | D | |  |
|  | 14 | | 83 | | M | |  | | Acute pancreatitis | | 10/5/6 | | - | | D | | D | |  |
|  | 15 | | 54 | | F | |  | | Acute leukemia | | 07/2/23 | | LU | | D | | D | |  |
|  | 16^b^ | | 67 | | M | |  | | IIP | | 07/1/5 | | LL | | D | | D | |  |
|  | 39 | | 67 | | M | |  | | Idiopathic thrombocytopenic purpura | | 07/10/11 | | RL | | N | | N | |  |
|  | 40 | | 39 | | M | |  | | Acute pancreatitis | | 07/9/18 | | - | | N | | N | |  |
|  | 41 | | 79 | | M | |  | | IIP | | 08/6/25 | | RM | | N | | N | |  |
|  | 42 | | 67 | | M | |  | | IIP | | 07/4/22 | | LU | | N | | N | |  |
|  | 43 | | 68 | | M | |  | | Acute myocardial infarction | | 11/10/14 | | LL | | D | | D | |  |
|  |  | |  | |  | |  | |  | |  | |  | |  | |  | |  |
| UIP | 17 | | 80 | | M | |  | | IIP | | 06/5/19 | | LL | | D | | D | |  |
|  | 18 | | 67 | | M | |  | | IIP | | 06/5/29 | | LL | | D | | D | |  |
|  | 19 | | 73 | | F | |  | | IIP | | 09/7/14 | | - | | D | | D | |  |
|  | 20 | | 81 | | M | |  | | Lung carcinoma | | 09/11/26 | | LL | | D | | D | |  |
|  | 44^a^ | | 82 | | M | |  | | Congestive heart failure | | 10/2/3 | | RU | | D | | D | |  |
|  | 45 | | 76 | | M | |  | | IIP | | 10/5/12 | | LL | | D | | D | |  |
|  | 46^c^ | | 65 | | F | |  | | IIP | | 08/5/13 | | RL | | D | | N | |  |
|  | 47 | | 57 | | M | |  | | Myocardial infarction | | 08/10/10 | | LL | | N | | D | |  |
|  | 48 | | 73 | | M | |  | | IIP | | 08/2/10 | | RL | | D | | D | |  |
|  | 49 | | 66 | | M | |  | | IIP | | 11/1/7 | | LU | | D | | D | |  |

^a, b^ and ^c^ Identical patients for each symbol.

IIP, idiopathic interstitial pneumonia; RU, right upper; RM, right middle; RL, right lower; LU, left upper; LL, left lower; -, not specified; D, detectable; N, not detectable
