# Supplementary figures and images for: Increased ectodomain shedding of cell adhesion molecule 1 as a cause of type II alveolar epithelial cell apoptosis in patients with idiopathic interstitial pneumonia
Source: Respir Res. 2015 Aug 1;16:90. doi: 10.1186/s12931-015-0255-x (PMC4531801; doi:10.1186/s12931-015-0255-x)

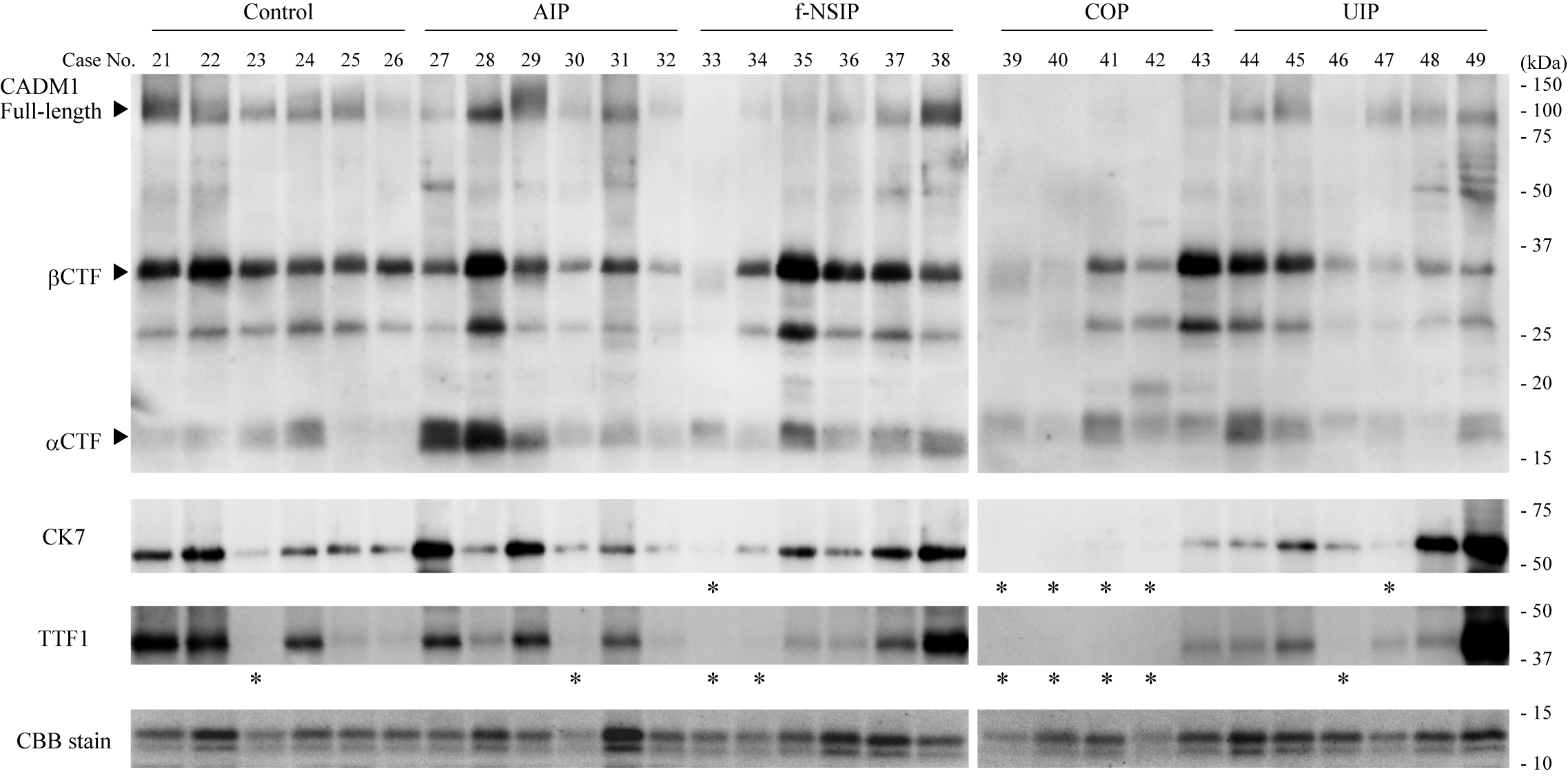

Supplement: Additional file 2: Figure S1. — Western blot analysis of CADM1 expression in IIP lungs. Protein was extracted from control and IIP lung sections and subjected to Western blot analyses using an anti-CADM1 antibody. Cases are numbered as in Additional file 1: Table S1. Arrowheads indicate bands corresponding to the full-length, αCTF, and βCTF forms of CADM1. The blot was reprobed with an anti-CK7 and anti-TTI1 antibody to estimate the number of epithelial cells and AECIIs, respectively. Asterisks indicate the samples that did not gave a clear immunoreactive band for CK7 or TTF1. After Western blot transfer, the gels were stained with CBB to indicate the amount of protein loading per lane. (TIFF 1277 kb) [file 12931_2015_255_MOESM2_ESM.tif]

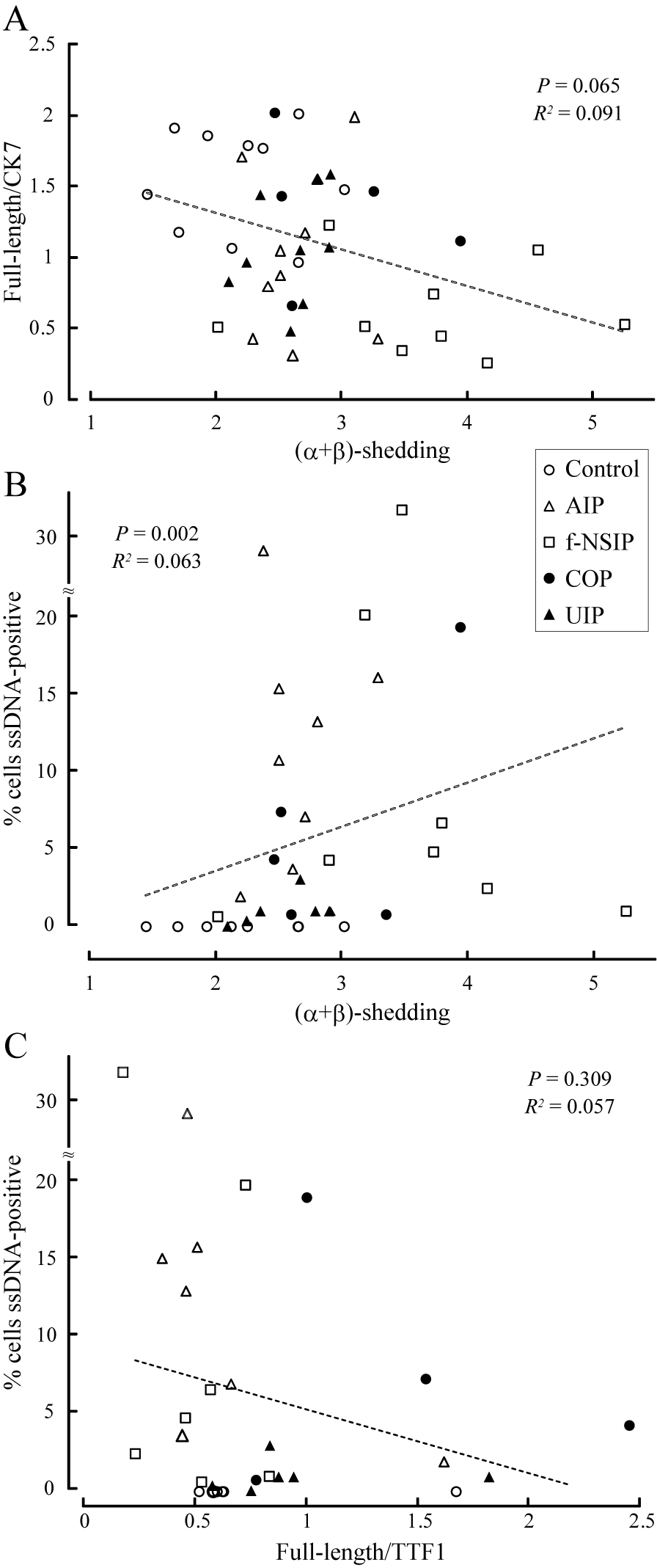

Supplement: Additional file 3: Figure S2. — Correlations of the full-length CADM1 level per epithelial cell or AECII apoptosis with (α + β)-shedding rate in IIP. (A) The full-length CADM1 level per epithelial cell with the CADM1 (α + β)-shedding rate are shown in a scatter plot. (B and C) The proportion of ssDNA-positive AECIIs with the CADM1 (α + β)-shedding rate (B) and the full-length CADM1 level normalized to TTF1 (C) are shown in scatter plots. In each graph A, B and C, the dot distribution approximates a linear function (dotted lines). Correlations and statistical significance were analyzed using Spearman’s rank test. R 2 and P-values are shown. (TIFF 208 kb) [file 12931_2015_255_MOESM3_ESM.tif]

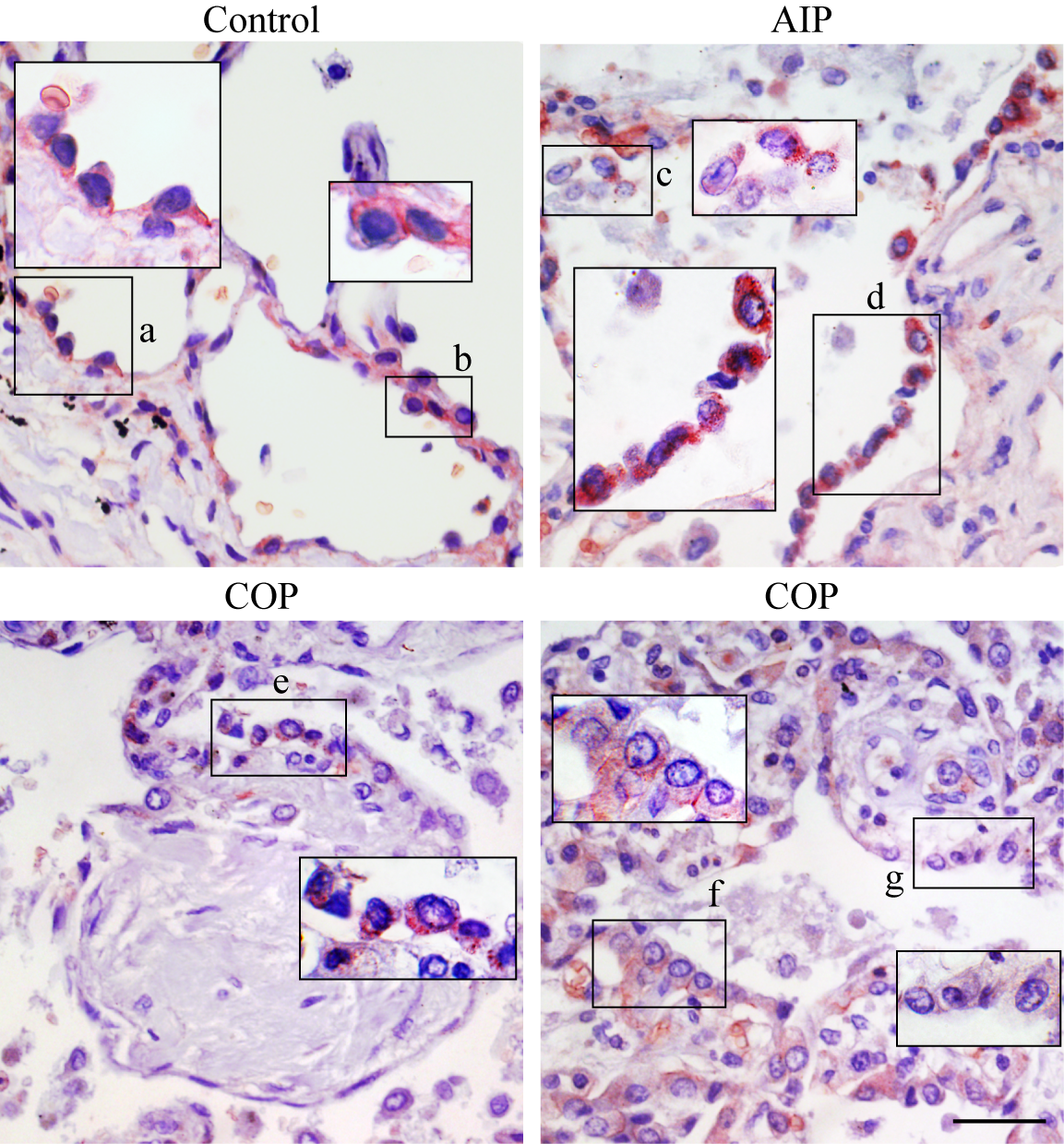

Supplement: Additional file 4: Figure S3. — Immunohistochemical analysis of CADM1 in IIP lungs. IIP lung sections were stained immunohistochemically with an anti-CADM1 antibody and counterstained with hematoxylin. Representative results for control lungs, AIP and COP are shown. Boxed areas are enlarged to depict different subcellular localizations of CADM1 in epithelial cells that are lining the alveolar lumen (a, b and f), detaching (e and g), or detached (c and d) from the alveolar wall. Bar = 50 μm. (TIFF 2572 kb) [file 12931_2015_255_MOESM4_ESM.tif]

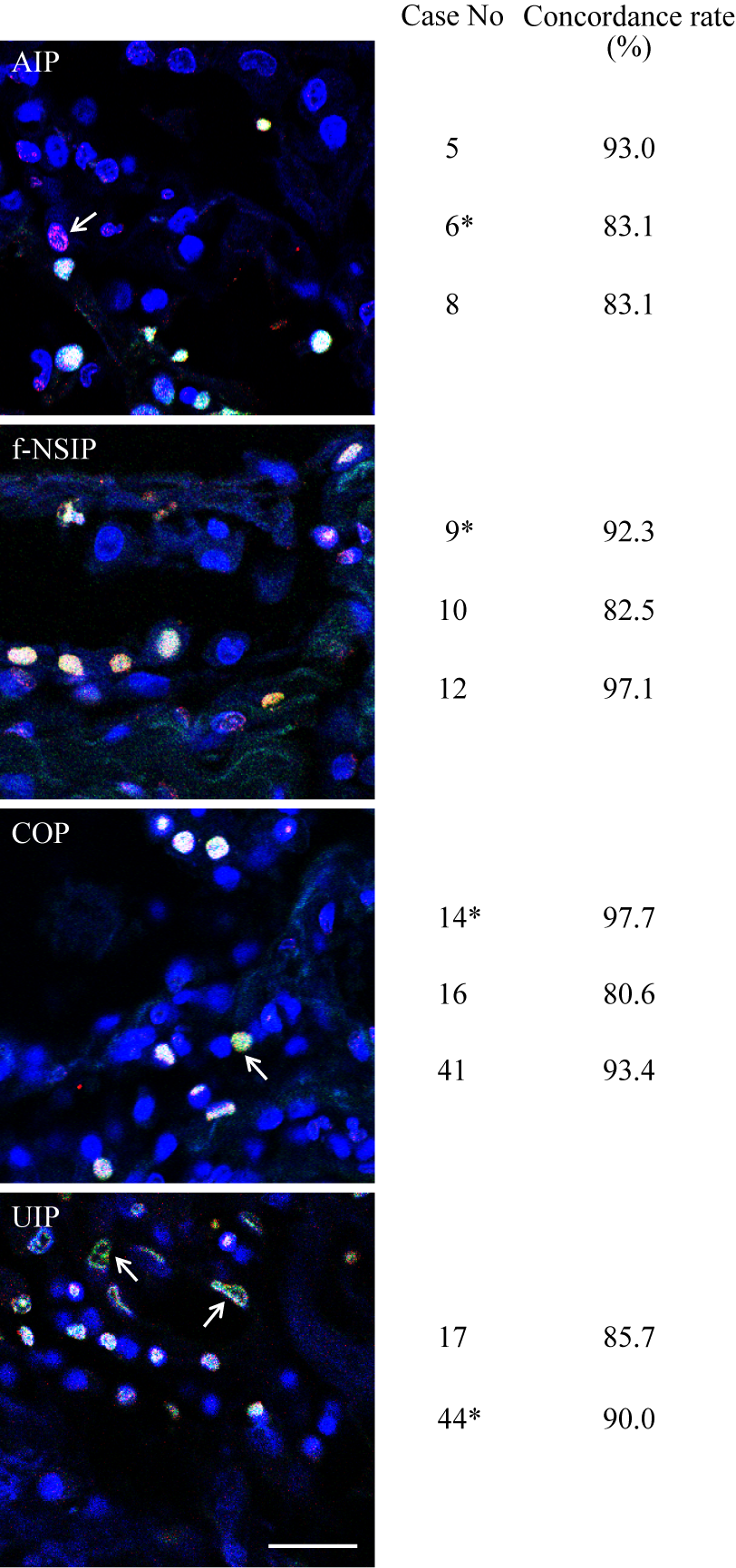

Supplement: Additional file 5: Figure S4. — Double staining of IIP lungs for two apoptosis markers, ssDNA and TUNEL. IIP lung sections from the cases indicated were stained serially using the TUNEL methods (green) and ssDNA immunofluorescence (red), and then reacted with DAPI (blue) to stain the nucleus. Representative results from the cases indicated by asterisks are shown as pictures where the three fluorescence images are merged. White signals indicate the nuclei double positive for ssDNA and TUNEL. Some nuclei are single positive for either marker depicted by arrows. The proportions of double-positive cells among ssDNA-positive AECIIs (concordance rate) are shown in the rightmost column. Bar = 50 μm. (TIFF 1437 kb) [file 12931_2015_255_MOESM5_ESM.tif]

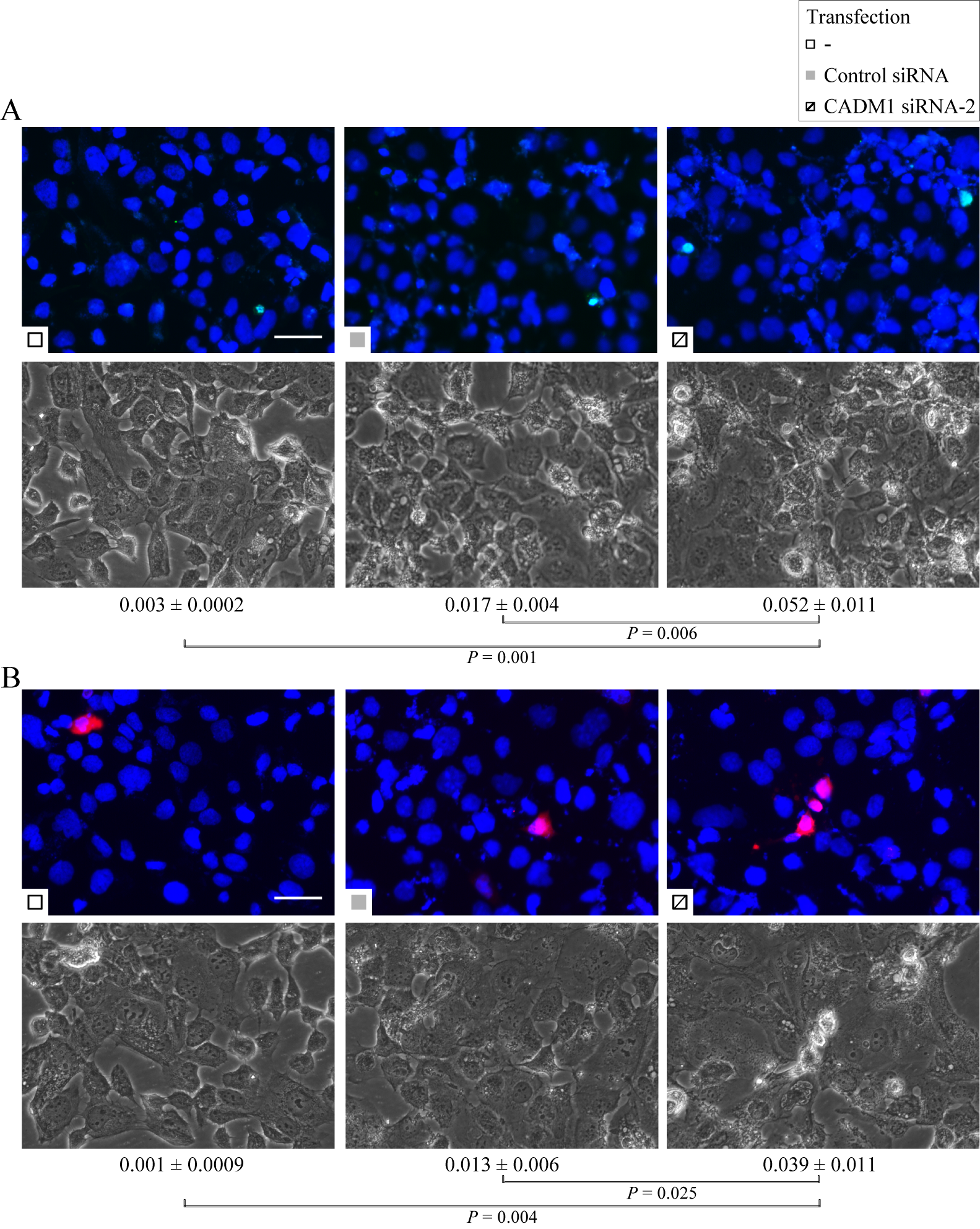

Supplement: Additional file 6: Figure S5. — Increased apoptosis of NCI-H441 cells transfected with a CADM1 siRNA-2 vector. (A) NCI-H441 cells were untreated or transfected with either control or CADM1 siRNA-2 vector, as described in Fig. 4, and then were analyzed by TUNEL assays. In the upper column, TUNEL (green) and DAPI (blue) fluorescent images were merged. The differential interference contrast images are shown on the lower. (B) Another set of NCI-H441 cells untreated and transfected with either control or CADM1 siRNA-2 vector was stained by immunofluorescence for cleaved caspase-3. In the upper column, cleaved caspase-3 (red) and DAPI (blue) fluorescent images were merged. The differential interference contrast images are shown on the lower. The mean proportions of TUNEL-positive cells and the SE were indicated under the panels. P-values ≤ 0.05 by Student’s t-test are shown. Bar = 50 μm. (TIFF 3101 kb) [file 12931_2015_255_MOESM6_ESM.tif]

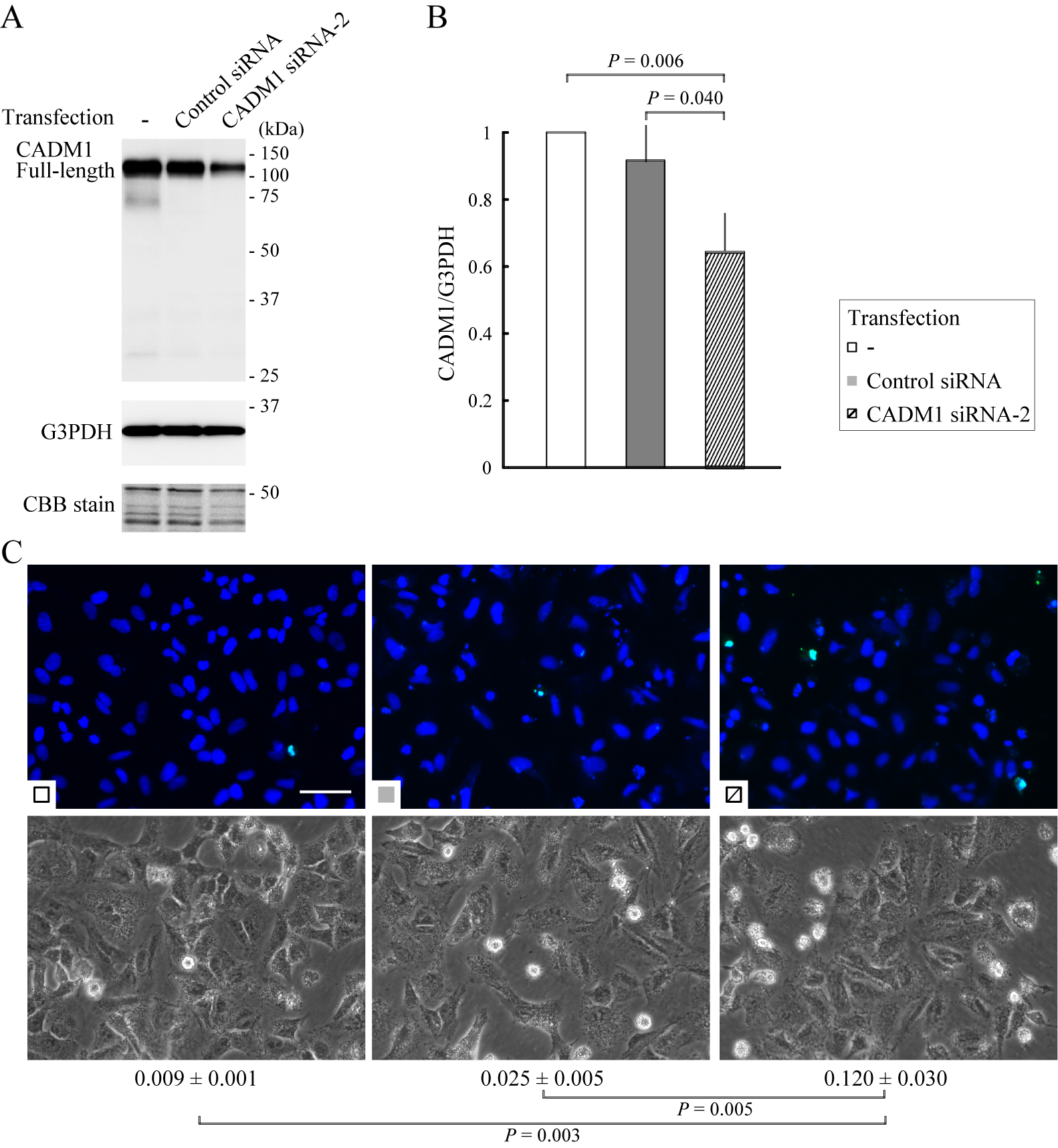

Supplement: Additional file 7: Figure S6. — Increased apoptosis of A549 cells transfected with an siRNA targeting CADM1. (A) A549 cells were left untreated (–) or transfected with control or CADM1-targeting siRNA (siRNA-2). After 48 h, CADM1 expression was examined by Western blot analyses. The blot was reprobed using an anti-G3PDH antibody to normalize full-length CADM1 levels to G3PDH. After Western blot transfer, the gels were stained with CBB to indicate the amount of protein loading per lane. (B) The relative CADM1 levels were further normalized to a value of 1 in untreated cells, to which the CADM1 levels in transfectants were then normalized. The mean ± SE full-length CADM1 levels were calculated from triplicate experiments for each cell type, and statistical significance was analyzed using Student’s t-test. P-values ≤ 0.05 are shown. (C) Another set of A549 cells untreated and transfected with either control or siRNA-2 vector was analyzed by TUNEL assays. In the upper column, TUNEL (green) and DAPI (blue) fluorescent images were merged. The differential interference contrast images are shown on the lower. Bar = 50 μm. The mean proportions of TUNEL-positive cells and the SE were indicated under the panels. P-values ≤ 0.05 by Student’s t-test are shown. (TIFF 1716 kb) [file 12931_2015_255_MOESM7_ESM.tif]
